# Retraction Note to: Natural product pectolinarigenin inhibits osteosarcoma growth and metastasis via SHP-1-mediated STAT3 signaling inhibition

**DOI:** 10.1038/s41419-021-03529-7

**Published:** 2021-03-15

**Authors:** Tao Zhang, Suoyuan Li, Jingjie Li, Fei Yin, Yingqi Hua, Zhouying Wang, Binhui Lin, Hongsheng Wang, Dongqing Zou, Zifei Zhou, Jing Xu, Chengqing Yi, Zhengdong Cai

**Affiliations:** 1 Department of Orthopaedics, Shanghai General Hospital, Shanghai Jiao Tong University School of Medicine, Shanghai, China; 2 The Institute of Cell Metabolism and Disease, Shanghai Key Laboratory of Pancreatic Cancer, Shanghai General Hospital, Shanghai Jiao Tong University School of Medicine, Shanghai, China

Retraction Note to: *Cell Death & Disease*

10.1038/cddis.2016.305 published online 13 October 2016

The authors previously corrected^1^ this article because of errors in Fig. 1e (the merge image of control group) and Fig. 5e (Pec. 50 mg/kg group). However, the authors have now noticed that the data shown in two panels in Fig. 4 are incorrect, namely Fig. 4a (adhesion assay) and Fig. 4b (invasion assay, pec 20 µM), and have therefore decided to retract this article. All authors agree with this retraction.
